# (Radio)Theranostic Patient Management in Oncology Exemplified by Neuroendocrine Neoplasms, Prostate Cancer, and Breast Cancer

**DOI:** 10.3390/ph13030039

**Published:** 2020-03-05

**Authors:** Irina Velikyan

**Affiliations:** Science for Life Laboratory, Department of Medicinal Chemistry, Uppsala University, 751 83 Uppsala, Sweden; irina.velikyan@akademiska.se

**Keywords:** (radio)theranostics, somatostatin, neuroendocrine neoplasms, prostate cancer, breast cancer, PSMA, PET, SPECT, gallium-68, lutetium-177

## Abstract

The role of nuclear medicine in the management of oncological patients has expanded during last two decades. The number of radiopharmaceuticals contributing to the realization of theranostics/radiotheranostics in the context of personalized medicine is increasing. This review is focused on the examples of targeted (radio)pharmaceuticals for the imaging and therapy of neuroendocrine neoplasms (NENs), prostate cancer, and breast cancer. These examples strongly demonstrate the tendency of nuclear medicine development towards personalized medicine.

## 1. Introduction

The number of nuclear medicine examinations and therapeutic procedures is increasing with acceleration worldwide reflecting the growing importance of the field in the modern healthcare system. The growth and expansion of nuclear medicine relies on the development and availability of radiopharmaceuticals. High demand for radiopharmaceuticals with biological activity specific for a certain disease yielded personalized patient treatment approaches, in particular theranostics using molecular imaging for the disease staging and prediction of the efficacy of specific therapeutic interventions on individual basis as well as for the monitoring response to the treatment. Molecular imaging in nuclear medicine is presented by positron emission tomography (PET) and single photon emission computed tomography (SPECT), and in combination with endoradiotherapy it can be defined as radiotheranostics [1].

Ideally the pre-therapeutic imaging (PET, SPECT) and subsequent endoradiotherapy should be conducted using the radioactive isotopes of the same chemical element, e.g., ^123^I/^124^I/^131^I [1]. However, the development of the respective radiopharmaceuticals is not always possible to achieve in practice. Metal radionuclides offer an advantage in terms of similarities in labelling chemistry, e.g., ^68^Ga(III), ^111^In(III), and ^177^Lu(III), while providing variation in the radiation mode relevant for both diagnostic imaging (PET, SPECT) and endoradiotherapy. Moreover, ^68^Ga(III), ^111^In(III), and ^177^Lu(III) can use the same chelator (DOTA) thus introducing the least possible difference in molecular structure and consequently specific target, e.g., receptor, binding properties.

**Figure 1:**
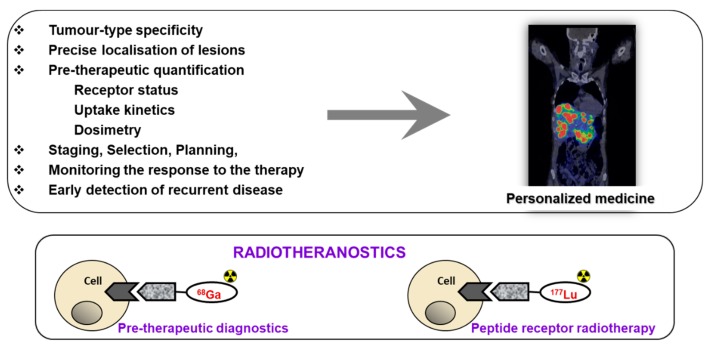
**Upper panel:** The targeted imaging in oncology provides tumor-type specific non-invasive diagnosis, precise localization of tumors and metastases that most importantly have the potential for pre-therapeutic quantification of receptor status, uptake kinetics and dosimetry that enables accurate therapy selection and planning, as well as monitoring response to the therapy resulting in personalized medicine. **Lower panel:** Some imaging and therapeutic radionuclides have similar coordination chemistry thus allowing for the radiotheranostic development wherein the pre-therapeutic imaging and radiotherapy are conducted with the same vector molecule exchanging the imaging and therapeutic radionuclides. Drawing presents the interaction of an agent, either imaging if labeled with, e.g., ^68^Ga (left) or radiotherapeutic if labeled with ^177^Lu (right), with the cell receptor. Reproduced from [8].

Receptor targeted chemo- and radiotherapeutics gain treatment precision and efficiency due to the prior quantification using molecular imaging for the stratification of the patients, adjustment of the therapeutic dose, and monitoring response (Figure 1). The treatment regimen depends on the stage of the disease at initial diagnosis and thus whole-body quantitative imaging reflecting heterogeneity of receptor expression and variability amongst patients is of utmost importance [2,3,4,5]. Further crucial advantage is possibility to monitor response to the therapy to introduce the treatment changes if necessary, as early as possible. Theranostics/radiotheranostics has strong potential not only for the optimized treatment but also for the exclusion of futile treatments that otherwise would cause unnecessary costs and patient distress. Apart from non-invasive imaging, radiation offers possibility of intraoperative detection for more accurate lesion resection.

Targeting specific biomarkers turns PET and SPECT imaging technologies into whole-body, non-invasive “biopsy”. It allows to overcome such disadvantages of a conventional biopsy such as sampling error, inability of taking multiple and repeated biopsies, inability to collect biopsies from certain areas, e.g., bone or brain, receptor expression heterogeneity with discordance of primary tumor and metastases as well as infection, hemorrhage, and patient discomfort. The variability in receptor density and subtype is a very crucial factor influencing the accuracy of diagnosis based on the pathological evaluation of a biopsy, and thus the imaging that reveals such variation in a single examination globally and quantitatively is of utmost important for individualized treatment [5,6]. 

The pioneer and most prominent example of the receptor targeted (radio)theranostics that has already been introduced into clinical practice is management of patients with neuroendocrine neoplasms (NENs) using somatostatin (SST) analogue based radiopharmaceuticals. Following the footsteps of SST receptor (SSTR) targeted (radio)theranostics, prostate specific membrane antigen (PSMA) targeting radiopharmaceuticals spread around the world with unprecedented acceleration. Two reporting and data system classifications for PSMA- and SSTR-targeted PET imaging have been introduced in order to navigate molecular imaging-guided treatment strategies [7]. Another current example of theranostics that evoked strong clinical interest was the management of breast cancer targeting human epidermal growth factor receptor type 2 (HER2) wherein the quantitative PET navigates the anti-HER2 targeting chemotherapy with antibody-based pharmaceuticals. This review demonstrates tendency of nuclear medicine development towards personalized medicine based on the abovementioned examples in the context of (radio)theranostics with its possibilities and challenges.

## 2. Targeting SSTR on Neuroendocrine Neoplasms

The clinical use of radiolabeled somatostatin analogues for imaging and radiotherapy has been accepted globally. Interestingly, the incidence of NENs increased from 1.09 to 6.98 per 100,000 individuals what might partly be explained by earlier detection and diagnosis due to availability of imaging technologies in clinical practice [9]. NENs are heterogeneous tumors and it is of utmost importance to identify patients who might benefit from the SST receptor (SSTR) targeted therapy [10]. PET/CT with ^68^Ga-labeled somatostatin ligand analogues ([^68^Ga]Ga-SST/PET) is recommended for the diagnosis, staging, and patient selection for endoradiotherapy [11]. Mono- and multi-center clinical trials demonstrated benefits of SSTR targeted pre-therapeutic imaging and radiotherapy in terms of patient management efficiency, efficacy, safety, and survival [12,13,14]. [^68^Ga]Ga-SST was found valuable not only for pre-operative assessment of resectable lesions and multiple unresectable lesions but also for intraoperative radio-guided resection [15,16]. [^68^Ga]Ga-SST/PET is also efficient tool for the scheduling of the treatment combining long-acting somatostatin analogue (SSA) therapy and endoradiotherapy [17] since the amount of the administered peptide influences the biodistribution pattern and might enhance lesion uptake while reducing uptake in the healthy tissue and organs with physiological expression of the target [2,18,19]. Guidelines for the standard care of NENs include radionuclide SSTR targeted imaging and therapy [20,21]. 

**Figure 2:**
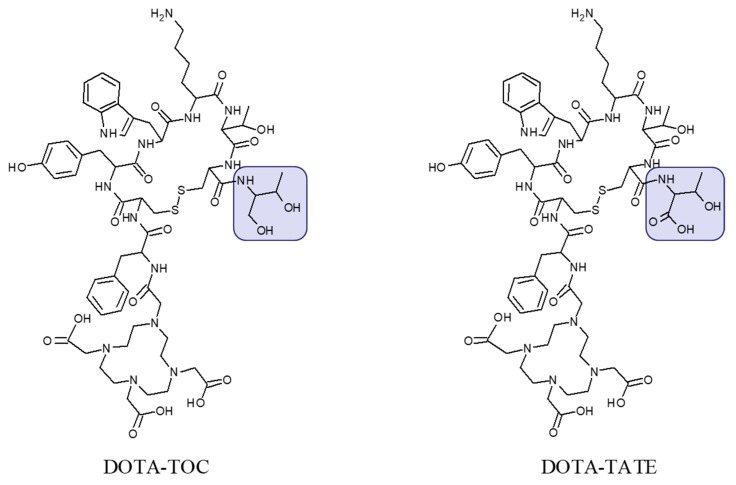
Chemical structure of DOTA-Tyr3-octreotide (DOTA-TOC) and DOTA-(Tyr3, Thr8)-octreotate (DOTA-TATE) presenting difference in C-terminal where the carboxyl group in threonine amino acid residue (DOTA-TATE) is exchanged to hydroxyl group (DOTA-TOC).

The most commonly used analogues are based on DOTA-Tyr^3^-octreotide (DOTA-TOC) and DOTA-(Tyr^3^, Thr^8^)-octreotate (DOTA-TATE) (Figure 2) labeled with ^68^Ga and ^177^Lu, respectively for PET imaging and endoradiotherapy. Advances in regulation, marketing of pharmaceutical grade ^68^Ge/^68^Ga generators, commercial availability of precursor peptides, radiopharmaceuticals and kits for the preparation of the radiopharmaceuticals under radiopharmacy conditions facilitate the dissemination of the technology. [^68^Ga]Ga-DOTA-TATE/[^68^Ga]Ga-DOTA-TOC and [^177^Lu]Lu-DOTA-TATE have been approved by Food and Drug Administration (FDA) and European Medicines Agency (EMA) for PET examination and treatment of SSTR positive gastroenteropancreatic NETs in adults. 

The [^68^Ga]Ga-SST/PET-CT has become the most promising non-invasive technique to study NENs and demonstrated superiority over such imaging agents as [^123^I]MIBG, [^11^C]-HTP, [^18^F]FDG, [^18^F]FDOPA, [^111^In]-pentetreotide, and [^99m^Tc]-SST analogues [22,23,24,25,26,27,28,29,30,31,32,33,34,35,36,37,38,39,40,41,42,43,44,45]. However, the combination of these imaging agents might provide higher diagnostic accuracy, e.g., [^18^F]FDG is used for measuring the tumor metabolic rate whereas [^68^Ga]Ga-SST provides information on SSTR expression guiding the biopsy [12,46,47,48,49,50,51,52,53]. Combination of a high SUV on [^68^Ga]Ga-SST/PET-CT and minor/no [^18^F]FDG uptake was associated with better prognosis [54].

Advantages in terms of sensitivity and detection rate of [^68^Ga]Ga-SST/PET-CT were demonstrated over MRI and CT [55,56,57,58,59,60]. Comparison of [^68^Ga]Ga-SST/PET-CT and [^68^Ga]Ga-SST/PET-MR showed similar PET image quality however uptake quantification was found more accurate on PET/CT and detection rate for bone metastases was higher [61,62,63]. However, these technologies can also be complementary [64,65,66].

The vast experience with [^68^Ga]Ga-SST analogues demonstrated necessity for accurate discrimination between cancerous and benign lesions, physiological and inflammatory uptake [23,67,68,69,70,71,72,73,74]. Studies on biodistribution and radiation dosimetry of [^68^Ga]Ga-SST analogues in patients and healthy volunteers revealed low total effective dose allowing multiple examinations per year and no immediate or delayed toxicity [40,75].

### Impact of SST Radiopharmaceuticals on Patient Treatment Management

Selection of patients who might benefit from endoradiotherapy [35,76,77,78,79,80,81] or are legible for surgery relies on accurate staging. The quantification of marginal differences between baseline and follow-up [^68^Ga]Ga-SST PET images require high accuracy. The (semi)-quantitative assessment of the response, using [^68^Ga]Ga-SST PET/CT, to the endoradiotherapy with ^177^Lu- or ^90^Y-based somatostatin analogues and re-staging of the disease have entered clinical practice. Patient selection, prognosis, prediction of absorbed dose for radiotherapy, and treatment response based on [^68^Ga]Ga-SST PET/CT have been performed using various parameters such as maximum standardized uptake value (SUV_max_), tumor-to-background SUV_max_ ratio (TBR), tumor-to-liver SUV_max_ ratio (TLR), tumor-to spleen SUV_max_ ratio (TSR), functional volume (FV), K-Patlak and Ki [60,76,82,83,84,85,86,87,88,89,90,91,92,93,94,95,96,97]. SUV_max_ cut-off of 16.4 was proposed for patient stratification for endoradiotherapy [84]. The cut-off of 15.0 was significantly associated with survival [54]. Inverse correlation between SUV_max_ and Ki-67 index indicates that [^68^Ga]Ga-SST PET reflects cell proliferation and helps guide disease management [98,99]. Nevertheless the criteria for the assessment are still to be refined [85] and standardized.

**Figure 3:**
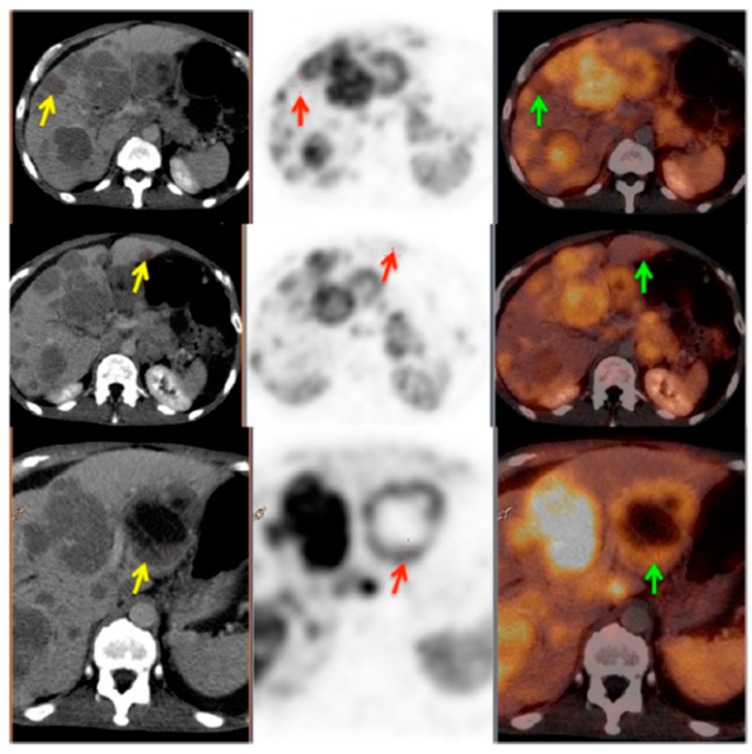
PET/CT image showing somatostatin receptor heterogeneity. Left panel: CT images; middle panel: PET images; right panel: PET/CT fused images. Yellow arrows show the CT lesions in liver; red and green arrows show corresponding lesions on PET and PET/CT fused images, respectively. This patient was characterized as heterogeneous as more than 50% of the target lesions showed heterogeneous somatostatin receptor (SSTR) expression. Reproduced from [104].

**Figure 4:**
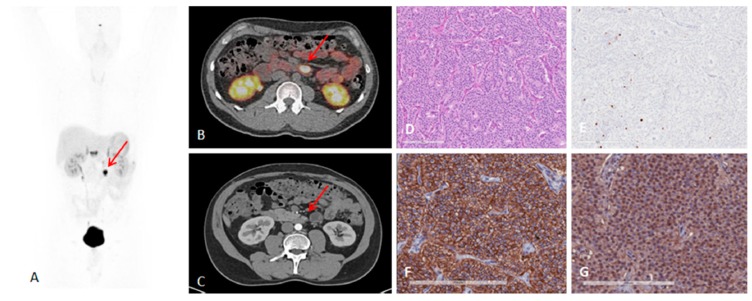
A 41-year-old man with metastatic lymph nodes on [^68^Ga]Ga-DOTA-TATE PET/CT. He had undergone previous surgery for pancreatic NET (patient 5). Mesenteric lymph node from primary pancreatic NET was found with probe with TBR of 3.4 for this 0.8 cm lesion (WHO grade 1). (**A**) [^68^Ga]Ga-DOTA-TATE PET maximum intensity projection image showing mesenteric/duodenal lesion in segment 3 (red arrow) and multiple retroperitoneal lymph nodes. (**B**) [^68^Ga]Ga-DOTA-TATE PET/CT image showing mesenteric mass with SUVmax of 72.8 (red arrow). (**C**) Arterial phase CT showing corresponding mesenteric mass (red arrow). (**D**) Representative hematoxylin and eosin staining of tumor showing NET in lymph node, WHO grade 1. (**E**) Representative immunohistochemistry for MIB-1 staining showing <2% of cells positive. (**F**) Immunohistochemistry for SSTR2 showing representative image for score 3 (membranous pattern of SSTR2 staining in >50% of tumor cells) (original magnification, ×20). (**G**) Immunohistochemistry for SSTR5 showing representative image of tumor with >10% of tumor cells positive (original magnification, ×20). Reproduced from [15].

Non-linear correlation between SUV and Ki indicated that SUV most likely did not reflect SSTR density accurately at higher SUVs [100]. While high correlation found between Ki and TBR indicated that the latter might be more accurate metrics than SUV for semi-quantitative assessment of [^68^Ga]Ga-SST lesion uptake and treatment response monitoring [101]. Total functional tumor volume (TFTV) measured on [^68^Ga]Ga-SST PET and computed by summing the volumes of all pathological foci was suggested as prognostic biomarker with cut-off of 13.8 cm^3^ [102]. Somatostatin receptor expressing tumor volume (SRETV), defined as tumor volume with higher [^68^Ga]Ga-SST uptake than 50% of SUV_max_ within the volume of interest for each lesion, demonstrated prognostic value of survival [103]. Visual assessment of SSTR heterogeneity on [^68^Ga]Ga-SST PET/CT images was found valuable for prediction and prognosis with heterogeneity leading to lower survival, even though it is difficult to quantify (Figure 3) [104]. [^68^Ga]Ga-SST PET uptake heterogeneity determined based on intratumoral textural features predicted endoradiotherapy outcome more accurately than SUV_max_ [105]. A threshold of 2.5–4.46 or higher for probe TBR was found a sensitive parameter for guided surgical resection (Figure 4) [15,16].

The tumor SUV_max_ reaches plateau 5 min post injection and remains unchanged within the range of 5–90 min [106] providing freedom of the examination logistics. However, it should be taken into consideration that the washout from the normal tissue and blood requires longer time influencing detection rate in the areas of high background uptake. 

**Figure 5:**
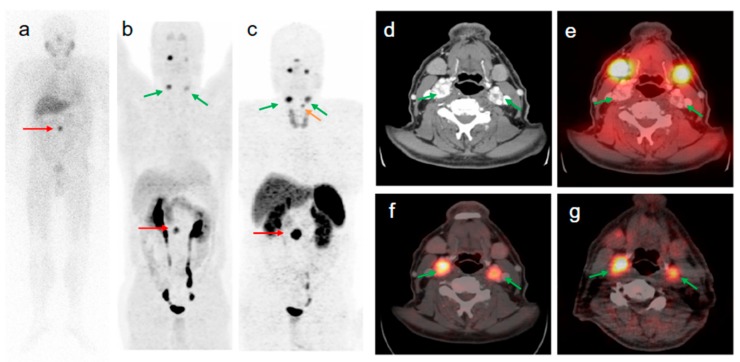
58-year old male patient with malignant extra-adrenal PGL. The intense focal [^123^I]MIBG uptake of the abdomen seen on anterior planar image ((**a**) red arrow) was confirmed by [^68^Ga]Ga-DOTA-TOC- and [^18^F]FDOPA PET/CT as being a soft tissue extra-adrenal PGL ((**b**,**c**) red arrows). After retrospective software-based image fusion of SPECT images with diagnostic CT images (**d**), symmetrical physiological [^123^I]MIBG uptake was observed in the parotid and submandibular glands (**a**,**e**). All verified head and neck lesions with focal [^68^Ga]Ga-DOTA-TOC ((**c**,**f**) green arrows) and [^18^F]FDOPA uptake ((**b**,**g**) green arrows) were [^123^I]MIBG negative (**a**,**e**). The small focus of [^68^Ga]Ga-DOTA-TOC uptake superior to the left upper lobe of the thyroid gland (c orange arrow) is a PGL lesion of the larynx (confirmed by diagnostic CT) which was negative in [^18^F]FDOPA PET (**b**). Compared to [^123^I]MIBG imaging (including SPECT/CT), [^68^Ga]Ga-DOTA-TOC PET/CT changed the tumor staging from non-malignant to metastatic disease. Reproduced from [43].

The fraction of patient treatments that were changed or adjusted based on [^68^Ga]Ga-SST/PET-CT examination was considerable and varied dependent on the patient cohort size and stratification [18,107,108]. The meta-analysis of clinical studies demonstrated that [^68^Ga]Ga-SST/PET-CT was vital for patient management leading to the regimen change in more than one third of patients (16%–71%) [109]. The treatment regimen was changed for 60% [110] and 50% [111] of the patients after [^68^Ga]Ga-SST/PET-CT. In the patient sub-group re-evaluated for recurrence, the treatment management was changed after [^68^Ga]Ga-SST/PET-CT in up to 25% of the patients [44]. Operative plans and diagnosis/management were adjusted, respectively in one-third and half of the patients after [^68^Ga]Ga-SST/PET-CT [112]. [^68^Ga]Ga-SST/PET-CT led to the treatment change in staggering 90.9% of patients with suspected recurrence [113]. [^68^Ga]Ga-SST/PET-CT changed the tumor staging from non-malignant to metastatic disease (Figure 5) [43].

**Figure 6:**
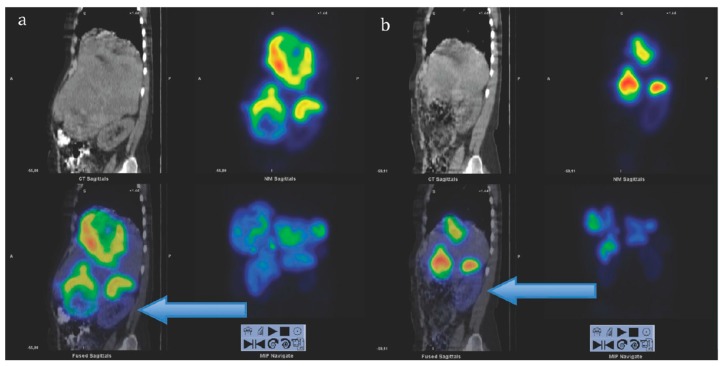
Sagittal views of single photon emission computed tomography (SPECT)-CT over the abdomen at the level of the right kidney, 24 hrs after infusion of 7.4 GBq of [^177^Lu]Lu-DOTA-octreotate. (**a**) Cycle 1, May 2010 (**b**) cycle 7, August 2011. Left upper corner in each image: Attenuation correction CT, right upper corner attenuation corrected SPECT, left lower corner fused SPECT-CT, right lower corner maximum intensity projection (MIP). Note the position of the right kidney (arrow) and tracer distribution within the tumors. Reproduced from [116].

As mentioned above the receptor density and type vary among lesions and within the same lesion contributing to the considerable variation in the individual characteristics of patients with similar clinical presentations. Consequently, optimization of endoradiotherapy in terms of administered radioactivity dose, number of cycles, and time delay between the cycles is required [114]. It could be achieved by individual pre-therapeutic quantitative dosimetry that would allow dose planning: (1) To avoid radiotoxicity to the essential radiosensitive and excretory organs, e.g., bone marrow and kidneys, to organs with physiological uptake of the radiopharmaceutical and healthy tissue surrounding lesions; (2) to avoid undertreatment in case of high tumor burden. ^177^Lu emits gamma particles that can be detected by SPECT for the dosimetry measurement and calculation, however not prior but during the therapy course. The [^177^Lu]Lu-DOTA-TATE dosimetry feasibility and impact on radiotherapy efficacy and outcome was demonstrated wherein the survival improved with increased treatment cycle number determined based on dosimetry (Figure 6) [115]. Fractionated [^177^Lu]Lu-DOTA-TATE therapy based on dosimetry also improved the treatment outcome [116]. 

As mentioned above [^177^Lu]Lu-DOTA-TATE SPECT dosimetry can earliest be performed after the first treatment cycle and it requires 3–4 examinations within one week making logistics complex and elevating costs. Quantification accuracy, higher spatial resolution, and dynamic scanning of PET are strong advantages over SPECT. [^68^Ga]Ga-SST/PET by a single examination and with minimal radiation dose to healthy organs would provide the required information prior to the radiotherapy with higher spatial resolution and quantification accuracy, thus allowing for better selection of patients and radiation dose planning. However, the straightforward use is precluded by the difference in physical half-lives of the radionuclides (68 min (^68^Ga) vs 6.71 d (^177^Lu)) and thus different pharmacokinetic time window. Kinetic modeling could provide a solution wherein the early distribution time points could be acquired by [^68^Ga]Ga-SST/PET with high accuracy [2] and possibly extrapolated to match therapeutic radionuclide time window providing higher resolution and quantification accuracy to predict absorbed doses to tumors and healthy organs. However, a prospective clinical study is needed to confirm this hypothesis. 

## 3. Targeting PSMA on Prostate Cancer

Prostate specific membrane antigen (PSMA) is a membrane bound protein overexpressed in prostate cancer, bladder carcinoma, schwannoma, and tumor neovasculature of many solid tumors [117]. The level of its expression is related to androgen independence, tumor aggressiveness, metastases, disease progression and recurrence, and the quantification of the upregulation would provide tool for accurate staging, prediction of aggressiveness and monitoring treatment response. 

**Figure 7:**
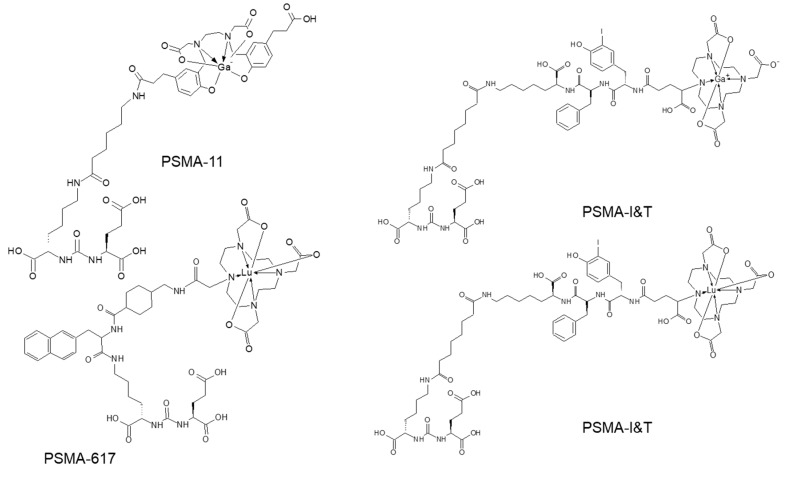
Molecular structures of prostate specific membrane antigen (PSMA) analogues based on Glu–urea–Lys motif.

Urea-based inhibitors of prostate specific membrane antigen representing low-molecular-weight peptidomimetics can image PSMA-expressing prostate tumors. Most analogues currently used in nuclear medicine are based on Glu–urea–Glu or Glu–urea–Lys motifs and it has been an explosive clinical use of the analogues labeled with various radionuclides (this review is focused on the analogues presented in Figure 7) [118]. The major radiometal-based analogues in clinical studies comprise HBED (N,N′-bis [2-hydroxy-5-(carboxyethyl)benzyl] ethylenediamine) and DOTA (1,4,7,10-tetraazcyclododecane-N,N′,N″,N‴-tetraacetic acid) chelators. 

### Impact of PSMA-Targeted Radiopharmaceuticals on Patient Treatment Management

In the first clinical trials, Glu-NH-CO-NH-Lys-(Ahx)-[^68^Ga(HBED-CC)] [119] demonstrated promising imaging results [120]. Since then numerous original research and review articles have pointed out that the PSMA imaging using PET/CT is a sensitive, specific, safe, efficient and reproducible diagnostic method allowing visualization of local disease, lymph node, bone, and visceral organ lesions with high detection rate, and it has positive predictive value [121,122,123,124,125,126,127,128]. Retrospective data analysis and prospective studies demonstrated the advantage of PSMA PET/CT compared to CT, MRI, and ^99m^Tc-MDP in terms of sensitivity and specificity that are crucial parameters for staging accuracy and treatment planning. It is relevant for initial staging [129], early detection of biochemical recurrence [130,131], and therapy planning and monitoring [132,133] particularly in patients with metastatic castration-resistant prostate cancer (mCRPC). Significant correlation between SUV_max_ on [^68^Ga]Ga-PSMA PET/CT and PSMA expression in primary prostate cancer, determined histopathologically, was found and cut-off for SUV_max_ of 3.15 to discriminate tumor from normal prostate was recommended [134]. Early imaging 5 min post injection was suggested for distinguishing lesions from urinary bladder [135]. 

**Figure 8:**
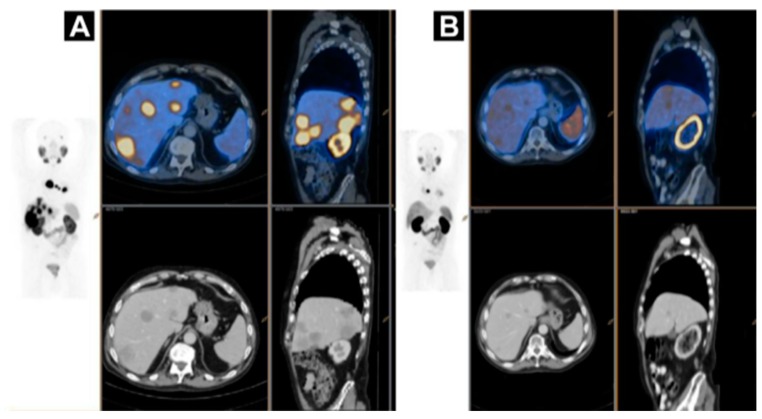
A case of homogenous treatment response (**A**) [^68^Ga]Ga-HBEDD PSMA-11/PET-CT: Highly PSMA-avid liver and mediastinal lymph nodal metastases (SUVmax 70) on screening (PSA 340 ng/mL). (**B**) [^68^Ga]Ga-HBEDD PSMA-11/PET-CT: 3 months after 4 cycles of [^177^Lu]Lu-PSMA therapy (PSA 1.5 ng/mL) exhibited marked biomarker and RECIST response with minimal residual PSMA activity in liver metastases and solitary PSMA-avid mediastinal lymph node. Reproduced from [146].

**Figure 9:**
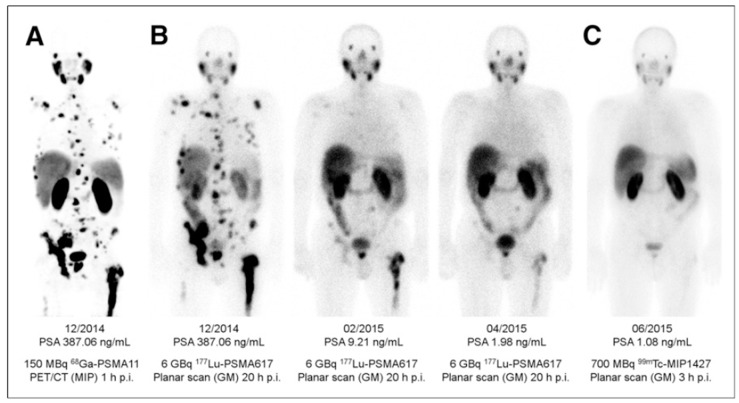
(**A**) Pre-therapeutic imaging using [^68^Ga]Ga-PSMA-11 PET/CT delivering highest resolution; (**B**) Co-emission of γ-rays by Lu-177 enables imaging during therapy with [^177^Lu]Lu-PSMA-617; (**C**) ^99m^Tc-PSMA scintigraphy has minimally less noise than posttherapy scanning and can be used for imaging follow-up in out-patient setting. GM 5 geometric mean; MIP 5 maximum-intensity projections; p.i. 5 after injection. Reproduced from [132].

PSMA PET has demonstrated strong impact on therapy planning and clinical decision making with treatment regimen adjustment in 27%–77% of patients [136,137,138,139,140,141,142,143,144]. The most frequently used pair in the context of radiotheranostics is [^68^Ga]Ga-PSMA-11 and [^177^Lu]Lu-PSMA-617 (Figure 7). Despite the structural difference and various radionuclides, it was demonstrated that [^68^Ga]Ga-PSMA-11 PET plays an important role in predicting treatment response to [^177^Lu]Lu-PSMA-617 and monitoring response for patient treatment management optimization [131,145,146,147,148]. Phase II prospective clinical trial demonstrated correlation of the treatment response with the uptake of [^68^Ga]Ga-PSMA-11 (Figure 8) [146]. Encouraging results in terms of safety, efficiency, response rate, toxicity, and reduction of pain have been demonstrated by clinical trials assessing the role of [^177^Lu]Lu-PSMA-617 in patients with mCRPC (Figure 9) [131,132,149,150]. 

Another Glu–urea–Lys motif based analogue, PSMA I&T, was labeled with ^68^Ga and ^177^Lu (Figure 7) respectively for the imaging and radiotherapy and demonstrated safe and effective radiopharmaceutical properties [151,152]. Both [^177^Lu]Lu-PSMA-617 and [^177^Lu]Lu-PSMA-I&T are found beneficial for patients in terms of survival and side effects [153,154]. The stratification of the patients, that would benefit from the endoradiotherapy, pre-therapeutic dosimetry, and treatment response monitoring were based on [^68^Ga]Ga-PSMA-PET/CT examination showing high correlation between PET/SUV_max_ and absorbed tumor dose of ^177^Lu analogue [155,156,157]. The radiation sensitive organs such as kidneys, bone marrow, and salivary glands require individual dosimetry assessment due to the inter-patient variance and for the subsequent administered therapeutic dose adjustment [150,156]. The European Association of Nuclear Medicine published guidelines for radionuclide therapy with [^177^Lu]Lu-labeled PSMA-ligand wherein PSMA-ligand/PET and [^18^F]FDG/PET are recommended for the selection of patients that would benefit from the therapy [158]. PSMA-radioguided surgery in prostate cancer may further improve the treatment outcome [123,159,160].

## 4. Targeting HER2 on Breast Cancer

**Figure 10:**
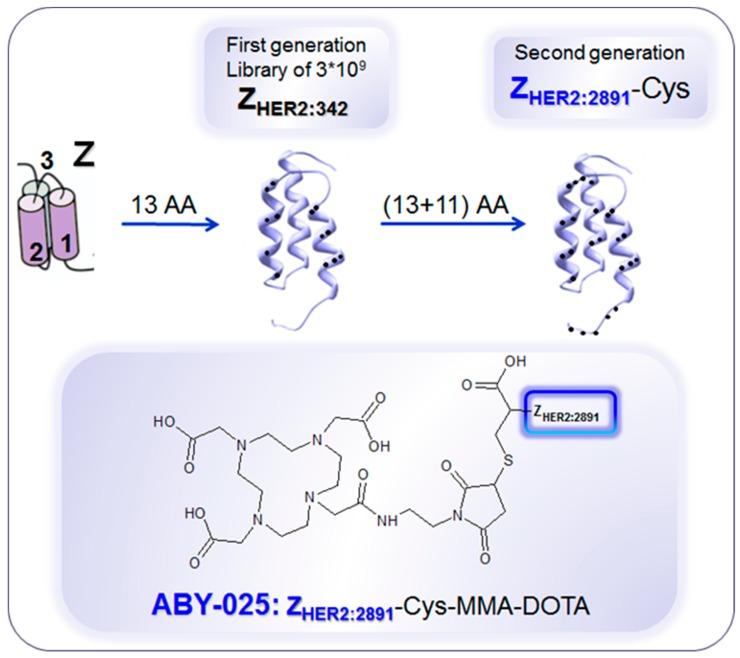
Development of anti-human epidermal growth factor receptor type 2 (HER2) Affibody^®^ molecules. The variation of 13 amino acids (AA) on the binding surface of a 3-helix peptide structure (Z) resulted in a combinatorial library containing billions of variants from which the 1st generation HER2-binding Affibody molecule, Z_HER2:342_, was selected. Further modification of the non-binding surface resulted in the second generation Affibody molecule, Z_HER2:2891_, with higher thermal stability and hydrophilicity, diminished background interactions with immunoglobulins and production flexibility by peptide synthesis or recombinant expression as well as fully retained in vitro and in vivo functionality. Z_HER2:2891_ was modified by addition of a unique terminal cysteine for site-specific conjugation to the bifunctional chelator, 1,4,7,10-tetraaza cyclododecane-1,4,7-tris-acetic acid-10-maleimidoethylacetamide (MMA-DOTA). Z_HER2:2891_–Cys binds selectively to HER2 with high affinity (K_D_: 60 pM). Reproduced from [183].

Pre-therapeutic imaging can be combined not only with endoradiotherapy, but also with chemotherapy yielding theranostic approach. HER2 is overexpressed in various malignant tumors, and particularly in 25% of breast cancer cases indicating poor survival [161,162,163,164,165,166]. Therapies based on antibodies and inhibitors targeting HER2 have revolutionized breast cancer treatment wherein the pre-therapeutic invasive biopsy for histopathological confirmation of sufficient HER2 expression (e.g., HercepTest^®^) for the patient selection and prediction of response is conducted [161,163,167,168,169,170]. However, heterogeneity of receptor expression within a lesion, and between the primary tumor and metastasis leads to such drawback with biopsy as sampling error. Moreover, it is highly invasive or not possible to perform sampling on bone and brain lesions as well as to collect samples from multiple lesions. Repeated sampling to monitor treatment response and receptor expression change over time is rarely possible in clinical practice [6,170,171,172]. Biopsy procedure causes patient distress and potential side effects such as infection and hemorrhage. The solution to provide whole-body HER2 receptor mapping and to overcome the biopsy drawbacks was found in radionuclide imaging, like in the cases of SSTR and PSMA. Various radiolabeled ligands based on antibodies, antibody fragments, EGFR natural ligand, Affibody^®^ molecules, and tyrosine kinase inhibitors targeting HER2 have been developed and studied pre-clinically and clinically [18,173,174,175,176]. Anti-HER2 Affibody^®^ molecule (Figure 10) presents advantages in terms of high affinity for HER2 receptors as well as favorable pharmacokinetics and clearance from non-target tissue [177,178,179,180,181]. The second generation Affibody^®^ molecule, ABY-025, binds selectively to HER2 receptors with picomolar affinity. Importantly, the binding site differs from that of trastuzumab and pertuzumab thus allowing imaging during the respective treatment [182].

### Impact of [^68^Ga]Ga-ABY-025 PET-CT on Patient Treatment Management

**Figure 11:**
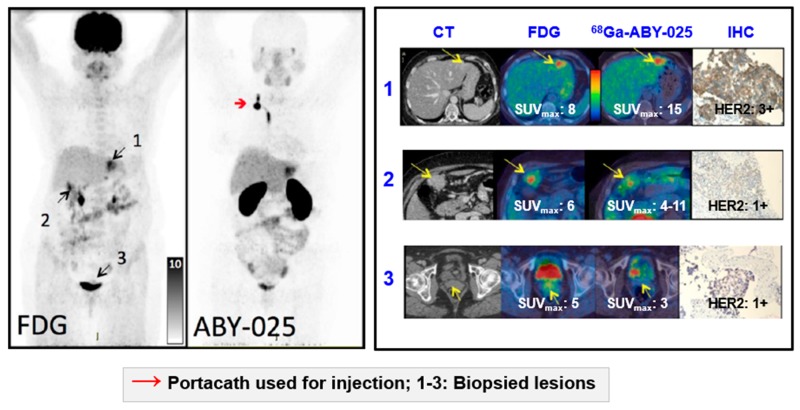
Based on the results from [^68^Ga]Ga-ABY-025 PET/CT, mixed expression of HER2 in metastatic breast cancer was seen in several patients and confirmed by biopsies in two. Patient 9 had HER2-negative primary tumor and was enrolled as negative control. [^18^F]FDG-PET/CT showed metastases in left liver lobe, peritoneal lymph nodes, and cervix of uterus. [^68^Ga]Ga-ABY-025 uptake was high in the liver metastasis, low in peritoneal metastases and absent in the cervical region (not shown). According to IHC, the liver finding was true positive and both other sites were true negative. Adopted from [5].

SPECT and PET imaging using anti-HER2 Affibody^®^ molecule [177,178,179,180,181,182] demonstrated the potential of safe, whole-body, and non-invasive “biopsy”, allowing receptor expression heterogeneity profiling (Figure 11), in clinical trials with ongoing multicenter Phase II/III one (NCT03655353) [5,183,184,185,186,187]. [^68^Ga]Ga-ABY-025 PET-CT presents advantages over [^111^In]In-ABY-025/SPECT/CT in terms of simpler logistics, higher resolution, higher detection rate, dynamic scanning, and accurate quantification [5,183] potentially allowing staging, prognosis, patient selection, quantification of the receptor expression and therapeutic drug dose estimation, early monitoring of the treatment response and resistance, residual disease, follow-up, and relapse.

**Figure 12:**
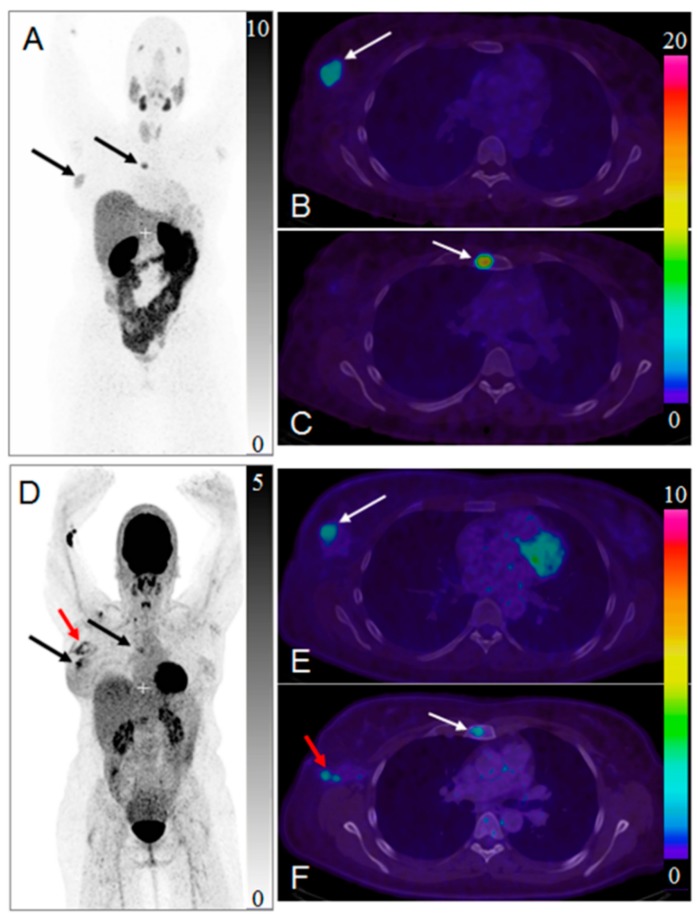
Maximum intensity projection positron emission tomography (PET) images of whole-body scan with [^68^Ga]Ga-ABY-025 ((**A**) 2 h) and [^18^F]FDG (**D**). Transaxial PET-CT fused images of the primary tumor ((**B**,**E**), respectively for [^68^Ga]Ga-ABY-025 (2 h) and [^18^F]FDG) and metastasis ((**C**,**F**), respectively for [^68^Ga]Ga-ABY-025 (2 h) and [^18^F]FDG). The black and white arrows indicate known tumor deposits. The red arrow (**D**,**F**) indicate post-surgical inflammation after biopsy wherein sentinel lymph node was tumor- and HER2-negative. Reproduced from [184].

HER2-targeted treatment was changed as a consequence of [^68^Ga]Ga-ABY-025 PET examination in 19% (*n* = 16) of patients [5]. Figure 12 presents a case wherein the prior IHC analysis of the primary tumor biopsy specimen showed a borderline expression of HER2 and consequently the treatment with Trastuzumab was not considered. However, subsequent [^68^Ga]Ga-ABY-025/PET-CT detected bone metastasis and primary tumor with high SUV_max_. The HER2-overexpression in the metastasis was confirmed by IHC. Consequently, the treatment regimen was significantly changed. Moreover, the false positive finding in the axilla by [^18^F]FDG/PET-CT (Figure 12D,F) was attributed to post-surgical inflammation. Given these examples it is difficult to overestimate importance of the targeting selectivity of [^68^Ga]Ga-ABY-025/PET-CT. Re-assessment of HER2 status is strongly encouraged due to high probability of the receptor conversion from positive to negative and vice versa [188]. Trastuzumab treatment was stopped after [^68^Ga]Ga-ABY-025/PET-CT examination that showed HER2 status conversion from positive to negative confirmed also by biopsy [5].

Extraordinary receptor shedding was observed after the start of HER2-targeted therapy wherein most probably the on-going treatment had executed a rapid cytotoxic impact on the metastases with HER2 debris leaking into the blood stream [186]. Liver biopsy after PET examination showed fibrosis and no sign of remaining cancer cells. Serum-HER2 level at the time of scanning was almost one hundred times higher than the normal upper limit. The high serum-HER2 level resulted in drastically altered organ distribution of [^68^Ga]Ga-ABY-025.

As in the case of SST analogues [2,17,18,19], the strong influence of the administered peptide amount on the organ distribution, tumor uptake and dosimetry was observed in the case of [^68^Ga]Ga-ABY-025 wherein the higher peptide dose radiopharmaceutical (427 µg vs 78 µg) presented higher detection rate and image contrast, more favorable organ distribution and lower effective and absorbed doses [5,183,187]. These examples strongly advocate for the importance of individualized therapeutical dose determination.

In order to facilitate standardized multicenter trials and to enable dissemination of this diagnostic methodology for routine clinical use, it is necessary to provide data evaluation methods that are independent on the variation of PET scanner characteristics. Intra-image normalization such as tumor-to-reference tissue ratio (T/R) was investigated and spleen was found the most accurate approach providing a simple and robust semi-quantification of HER2 expression [186]. The spleen T/R ratio met the selection criteria such as correlation with biopsy analysis results, low variation of radioactivity uptake, and low probability of hosting metastases from breast cancer on spleen. The suggested cut-off for the discrimination between HER2-positive and HER2-negative lesions was set to 6.5. Another crucial aspect for [^68^Ga]Ga-ABY-025 PET-CT technology worldwide spreading is production and availability of the radiopharmaceutical [184]. The automated production procedure is currently used in the ongoing phase II/III study, aiming to validate the use of [^68^Ga]Ga-ABY-025/PET-CT for non-invasive assessment of HER2-status in breast cancers in a multicenter setting (ClinicalTrials.gov: NCT03655353).

**Figure 13:**
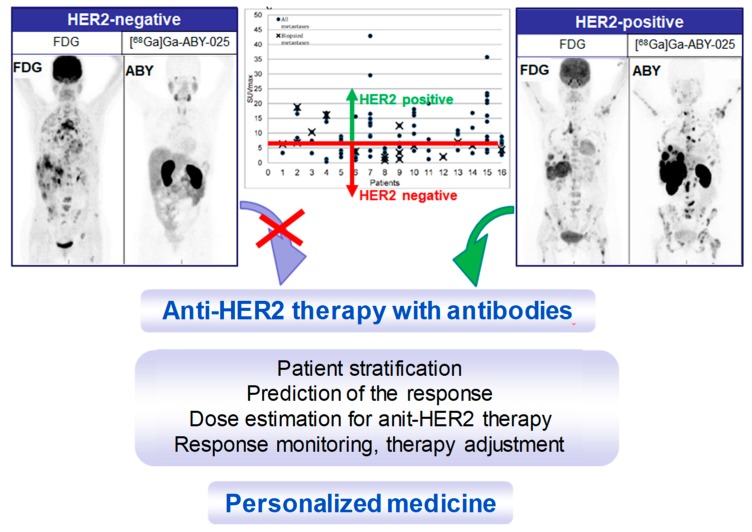
Maximum intensity projection PET images from two studied patients with wide-spread metastatic breast cancer. Left panel: HER2-negative. Right panel: HER2-positive. All images are normalized to SUV 10. Darker colors indicate higher uptake. The graph presents the range of [^68^Ga]Ga-ABY-025 SUV_max_ in all nodular metastases (black dots) identified using [^18^F]FDG-PET/CT in all 16 patients undergoing [^68^Ga]Ga-ABY-025 PET/CT at 2 h after injection of tracer with high peptide content. Crosses symbolize biopsied metastases. The red line indicates a proposed threshold at SUV_max_ = 6 for discriminating HER2-positive and HER2-negative metastases. Adopted from [5].

Reliable whole-body, quantitative assessment of HER2-receptor expression is crucial in order to identify patients with HER2-positive tumors that can benefit from HER2 targeted treatments (Figure 13). It is as important to avoid unnecessary cost and potential risk of serious adverse effects related to the treatment of patients with HER2-negative tumors. [^68^Ga]Ga-ABY-025 PET/CT has potential for therapy planning and treatment response monitoring, enabling adjustment of the treatment very early in the process.

## 5. Conclusions

These examples of NENs, prostate cancer, and breast cancer management using targeted imaging and (radio)therapy have proven the concept of (radio)theranostics for clinical practice valid. Numerous publications report on the alteration of treatment regimen based on radionuclide imaging that provides non-invasive, whole body mapping of the specific target in a single examination that can be safely repeated multiple times for monitoring treatment response and disease progression. Pre-therapeutic determination of the absorbed doses to normal organs and lesions is essential for treatment planning. However, the use of short-lived radionuclides for the prediction of dosimetry for long-lived therapeutic radionuclides presents challenge and therapeutic dose planning based on the receptor expression quantification awaits prospective clinical studies to prove the concept.

Nuclear medicine is becoming an important component in personalized patient treatment. Dissemination of the (radio)theranostic technology requires standardization and harmonization of the clinical protocols and data evaluation strategies, as well as accessibility and regulatory approval of radiopharmaceuticals.
